# Supplementary material for: The Role of Transthoracic Echocardiography for Assessment of Mortality in Patients with Carcinoid Heart Disease Undergoing Valve Replacement
Source: Cancers (Basel). 2023 Mar 21;15(6):1875. doi: 10.3390/cancers15061875 (PMC10046658; doi:10.3390/cancers15061875)
Supplement: Supplementary file 1 [file cancers-15-01875-s001.zip › cancers-2259761-supplementary.pdf]

Supplementary Materials: The Role of Transthoracic Echocardiography for Assessment of Mortality in Patients with Carcinoid Heart Disease Undergoing Valve Replacement

Abigail Brooke, Sasha Porter-Bent, James Hodson, Raheel Ahmad, Tessa Oelofse, Harjot Singh,Tahir Shah, Ahmed Ashoub, Stephen Rooney and Richard P. Steeds

Table S1. Pre-operative right heart catheterization parameters.

Parameter	*N*	*Average*	*Upper Limit of Normal Range*	*Above Normal Range**
Mean RA Pressure (mmHg)	*32*	*10 ± 5*	*5 mmHg*	*25 (78%)*
RV Pressure (mmHg)				
Systolic	*34*	*29 (24-34)*	*30 mmHg*	*12 (35%)*
Diastolic	*30*	*1 (0-4)*	*8 mmHg*	*2 (7%)*
RVEDP (mmHg)	*15*	*8 (4-12)*	*8 mmHg*	*6 (40%)*
PA Pressure (mmHg)				
Systolic	*39*	*23 (18-28)*	*30 mmHg*	*5 (13%)*
Diastolic	*38*	*7 (4-9)*	*12 mmHg*	*4 (11%)*
Mean	*39*	*14 (11-16)*	*15 mmHg*	*12 (31%)*
PCWP (mmHg)	*33*	*9 (7-13)*	*12 mmHg*	*9 (27%)*

Averages are reported as mean ± SD, or as median (IQR), as applicable. *The N (%) of patients with values greater than the stated upper limit of the normal range.
